# A Nucleoside Anticancer Drug, 1-(3-C-Ethynyl-β-D-Ribo-Pentofuranosyl)Cytosine, Induces Depth-Dependent Enhancement of Tumor Cell Death in Spread-Out Bragg Peak (SOBP) of Proton Beam

**DOI:** 10.1371/journal.pone.0166848

**Published:** 2016-11-22

**Authors:** Kenichiro Maeda, Hironobu Yasui, Tohru Yamamori, Taeko Matsuura, Seishin Takao, Motofumi Suzuki, Akira Matsuda, Osamu Inanami, Hiroki Shirato

**Affiliations:** 1 Department of Radiation Medicine, Graduate School of Medicine, Hokkaido University, Kita 15 Nishi 7, Kita-ku, Sapporo, Hokkaido, 060–8638, Japan; 2 Laboratory of Radiation Biology, Department of Environmental Veterinary Sciences, Graduate School of Veterinary Medicine, Hokkaido University, Kita 18 Nishi 9, Kita-ku, Sapporo, Hokkaido, 060–0818, Japan; 3 Division of Quantum Science and Engineering, Faculty of Engineering, Hokkaido University, Kita 13 Nishi 8, Kita-ku, Sapporo, Hokkaido, 060–8628, Japan; 4 Proton Beam Therapy Center, Hokkaido University Hospital, Kita 14 Nishi 5, Kita-ku, Sapporo, Hokkaido, 060–8648, Japan; 5 Graduate School of Pharmaceutical Sciences, Hokkaido University, Kita-12 Nishi-6, Kita-ku, Sapporo, Hokkaido, 060–0812, Japan; 6 Global Institution for Collaborative Research and Education, Hokkaido University, Kita 14 Nishi 5, Sapporo, Hokkaido, 060–8648, Japan; Istituto di Genetica Molecolare, ITALY

## Abstract

The effect of 1-(3-C-ethynyl-β-D-ribo-pentofuranosyl)cytosine (ECyd) on proton-induced cell death was evaluated in human lung carcinoma cell line A549 and Chinese hamster fibroblast cell line V79 to enhance relative biological effectiveness (RBE) within the spread-out Bragg peak (SOBP) of proton beams. Treatment with ECyd significantly enhanced the proton-induced loss of clonogenicity and increased senescence at the center, but not at the distal edge of SOBP. The p53-binding protein 1 foci formation assay showed that ECyd decelerated the rate of DNA double-strand break (DSB) repair at the center, but not the distal region of SOBP, suggesting that the ECyd-induced enhancement of proton-induced cell death is partially associated with the inhibition of DSB repair. This study demonstrated that ECyd enhances proton-induced cell killing at all positions of SOBP, except for the distal region and minimizes the site-dependent differences in RBE within SOBP. Thus, ECyd is a unique radiosensitizer for proton therapy that may be useful because it levels the biological dose within SOBP, which improves tumor control and reduces the risk of adverse effects at the distal edge of SOBP.

## Introduction

Proton beams have a characteristic depth-dose profile in which the physical dose is almost constant until near the end of the range, and then there is a sharp increase termed a Bragg peak. A region of high physical dose that can cover the volume of a tumor can be achieved using a spread-out Bragg peak (SOBP), which can be created using passive scattering or scanning techniques. This feature of proton beams enables the radiologist to deposit a high physical dose into a deep-seated tumor [1]. Proton therapy has been widely utilized for tumor therapy [2]. Recently, precise evaluation of relative biological effectiveness (RBE; the ratio of the absorbed dose of the particle beam to the absorbed dose of 250 kV X-rays or ^60^Co gamma-rays required to produce the same biological effect) has been reported for several positions in SOBP of passive scattering proton beams [3–7] and scanning proton pencil beams [8]. Matsumoto *et al*. [7] and our previous study [8] reported that the RBE value at the center of SOBP was 1.1–1.2, whereas the RBE value at the distal region of SOBP reached approximately 2.0 at the maximum, indicating that the RBE value in a particular region of SOBP depends upon the depth of that region within SOBP. However, when planning proton therapy in the clinic, 1.1 is used as the RBE for the entire SOBP for convenience.

Recently, proton therapy for unresectable, locally advanced non-small cell lung cancer NSCLC [9] and stage III–IVB tongue cancer [10] is performed with concurrent chemotherapy, such as cisplatin, to enhance RBE within the central region of SOBP of the proton beam. These clinical trials revealed that combination therapy has an acceptable toxicity profile and induces elongation of the survival time of patients. Furthermore, high-Z gold (Au) nanoparticles were reported to enhance proton-induced cell killing in DU-146 [11], EMT-6 and CT26 cells [12] due to a local dose enhancement. These data indicate that chemical agents and Au nanoparticles enhance RBE of proton beams. However, no information is available regarding chemical regents that can induce radiosensitization at multiple positions within SOBP. Therefore, to establish effective protocols that combine proton therapy with a chemical reagent, it is important to evaluate the effect of the chemical reagent on RBE of the proton beam and the biological dose (RBE × physical dose), at multiple positions within SOBP, including the edge regions.

The antitumor cytidine analogue 1-(3-C-ethynyl-β-D-ribo-pentofuranosyl)cytosine (ECyd; S1 Fig) has been shown to possess potent cytotoxic and antitumor activities in preclinical therapeutic models [13]. After its uptake into cells, ECyd immediately undergoes phosphorylation to its 5ʹ- triphosphate form, which strongly inhibits RNA polymerase, thereby inhibiting RNA synthesis [14, 15]. Our previous reports have demonstrated that sub-lethal doses of ECyd synergistically sensitizes X-ray-induced cell killing in various tumor cell lines through the downregulation of DNA repair enzymes, check-point-related proteins, and anti-apoptotic proteins [16–18]. ECyd also enhances X-ray-induced tumor growth delay in murine transplanted tumors [16]. If ECyd enhances proton-induced cell killing, as well as X-ray-induced cell killing, ECyd may be a useful radiosensitizer for proton therapy. In this study, we evaluated the effect of ECyd on proton beam-induced tumor cell killing at multiple positions within SOBP.

## Materials and Methods

### Cell culture

Human lung carcinoma cell line A549 and Chinese hamster fibroblast cell line V79 were purchased from RIKEN Cell Bank and grown in RPMI1640 medium (Thermo Fisher Scientific Inc., Waltham, MA) and α−MEM medium (Thermo Fisher Scientific Inc.), respectively, supplemented with 10% fetal bovine serum (BioWest, Nuaillé, France) at 37°C in an atmosphere containing 5% CO_2_.

### Proton irradiation and drug treatment

**Fig 1:**
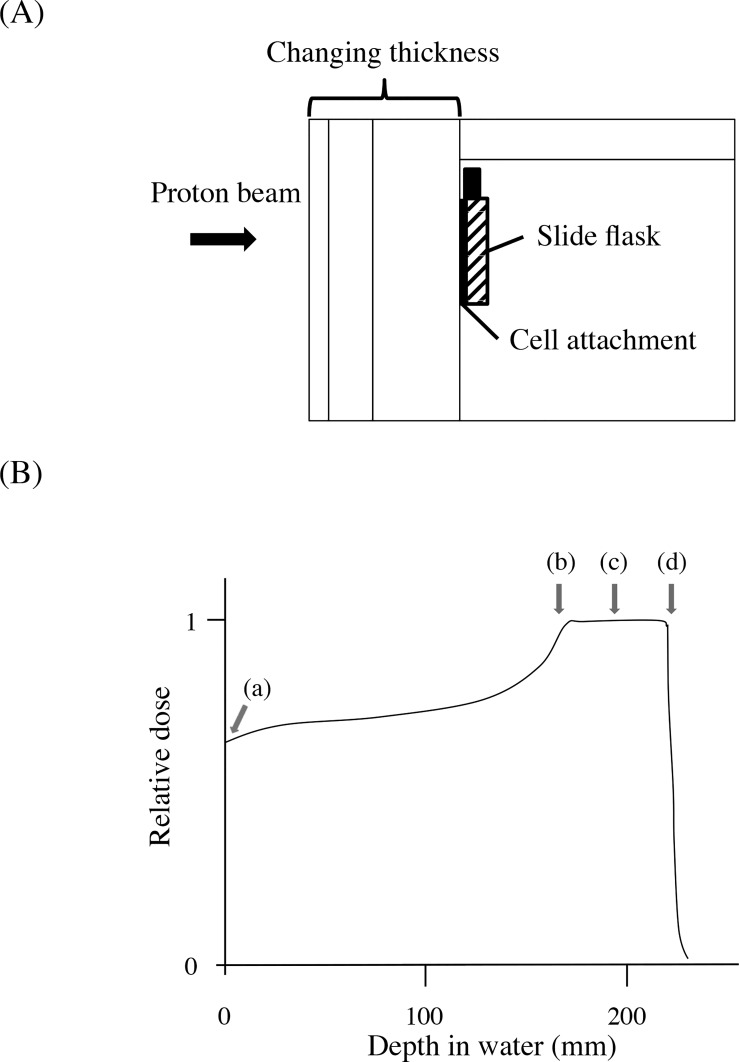
Irradiation phantom and cell position. (A) Schematic of the polyethylene phantom for cell irradiation. This phantom enables the thickness of the block in front of the flask to be changed (water equivalent ratio = 1.03, ρ = 0.98 g/cm^3^). (B) Positions of cell irradiation. The spread-out Bragg peak (SOBP) width was 6 cm, the energy range was from 156.7 MeV to 182.8 MeV, and the field size was 10 × 10 cm^2^. The isocenter plane was matched with the center of SOBP. (a) 5-mm depth from the primary plane, (b) proximal 95% physical dose point compared with the center of SOBP, (c) the center of SOBP, (d) distal 95% physical dose point compared with the center of SOBP. The depths corresponding to these positions are shown in Table 1.

ECyd was synthesized as described elsewhere [13]. Tumor cells were attached to a chamber slide flask (Lab-Tek™ SlideFlask 170920, Thermo Scientific/Nunc, Penfield, NY) at 1.8 × 10^6^ cells/flask and treated with ECyd at concentrations of 0.1 and 0.4 μM for A549 and V79 cells, respectively, 1 h before irradiation. Proton irradiation was performed at the Hokkaido University Hospital Proton Therapy Center. A high-density polyethylene block was used to position the cells during proton irradiation, (Fig 1A). As shown in Fig 1B, proton beam irradiation was performed at the following four points: (a) at a depth of 5 mm from the primary plane, (b) at the proximal 95% physical dose point from the center of the SOBP, (c) at the center of the SOBP, (d) at the distal 95% physical dose point from the center of SOBP (the distal edge).

### Clonogenic survival assay

Immediately after irradiation, cells were trypsinized and harvested. Cell suspensions were diluted and the proper number of cells was seeded in 6-cm dishes and cultured for 23 h using medium with or without ECyd. Then, the cells were washed twice with PBS and incubated with fresh medium. After incubation for 13 days (A549 cells) or 6 days (V79 cells), colonies were fixed with methanol and stained with Giemsa solution. Colonies containing more than 50 cells were scored as surviving cells. The surviving fraction at each dose was calculated with respect to the plating efficiency of the non-irradiated control and plotted at each physical dose. Survival curves were fitted using the linear-quadratic (LQ) model: SF = exp(-αD-βD^2^), where SF is the surviving fraction and D is the physical dose. Each experiment was performed at least three times.

### Senescence-associated β-galactosidase staining and quantification of senescent cells

The Senescence β-Galactosidase Staining Kit (Cell Signaling Technology, Danvers, MA, USA) was used to perform β-galactosidase staining. Cells were trypsinized and harvested immediately after irradiation with 9 Gy of proton irradiation and/or treatment with ECyd. Forty thousand cells were seeded in 3.5-cm dishes and cultured for 23 h in medium with or without ECyd. Then, the cells were washed twice with PBS and incubated with fresh medium. Senescent cells were stained according to the manufacturer’s protocol. The percentages of β-galactosidase-positive cells were determined by scoring at least 200 cells per sample at 20× magnification using a BZ-9000 inverted microscope (Keyence, Osaka, Japan). Three independent experiments were performed.

### Immunofluorescence staining for p53-binding protein 1, gamma-H2AX and mediator of DNA damage checkpoint protein 1

At the indicated times after proton irradiation (1.5 Gy) with or without ECyd treatment, cells on a chamber slide flask were fixed with 4% paraformaldehyde in PBS for 30 min at room temperature. After being permeabilized with PBS containing 0.5% Triton X-100 for 5 min at 4°C, cells were blocked by treatment with PBS containing 6% goat serum for 30 min at room temperature. The blocked cells were incubated with a rabbit anti- p53-binding protein 1 (53BP1) antibody (Abcam Inc., Cambridge, CA) at 1:1000 dilution, a rabbit Anti-gamma H2AX (γH2AX) antibody (Abcam Inc., Cambridge, CA) at 1:1000 dilution and a rabbit Anti-mediator of DNA damage checkpoint protein 1 (MDC1) antibody (Abcam Inc., Cambridge, CA) at 1: 100 dilution in 3% goat serum overnight at 4°C and then incubated in the dark with an Alexa Fluor 488-conjugated anti-rabbit secondary antibody (Abcam Inc., Cambridge, CA) at a 1:1000 dilution for 90 min. After incubation, they were counterstained with 300 nM 4ʹ,6 ʹ-diamidino-2-phenylindole (DAPI; Thermo Fisher Scientific Inc., Waltham, MA) for 5 min at room temperature and mounted with Prolong Gold antifade reagent (Thermo Fisher Scientific Inc., Waltham, MA). Fluorescence microscopic analysis was performed using an Olympus BX50 microscope (Olympus, Tokyo, Japan) with reflected light fluorescence, and foci were counted using Image J software (National Institutes of Health, Bethesda, MD). Three independent experiments were performed. An experiment was performed using X-rays (200 kV, 20 mA, 0.91 Gy/min) instead of proton beams as the irradiation source to provide a reference for low linear energy transfer (LET) irradiation.

### Calculation of dose-averaged LET

The dose-averaged LET (LET_d_) has been calculated for different ion beams because it can be used as a meaningful index of biological effectiveness, or to calculate RBE [19–21]. In this study, LET_d_ was calculated for all positions of cell irradiation using the analytical LET_d_ calculation approach described by Wilkens *et al* [22]. Briefly, LET_d_ distribution for each of the initial Bragg peaks composing SOBP was derived. Then, the individual LET_d_ distributions were superimposed using the weighting factors derived from the in-house simulation tools and treatment planning system VQA (Hitachi, Tokyo, Japan) used at the Hokkaido University Hospital Proton Therapy Center. LET_d_ values were calculated at four positions: (a) at a 5-mm depth from the primary plane, (b) at the proximal 95% physical dose point, compared with the center of SOBP, (c) at the center of SOBP and (d) at the distal 95% physical dose point, compared with the center of SOBP. These positions are depicted in Fig 1B.

### Statistical analysis

All results are expressed as the mean ± standard deviation. Differences between groups were analyzed using the Mann–Whitney U test, and those resulting in *P* values <0.05 were considered to be statistically significant.

## Results

### ECyd enhanced proton-induced cell death in a depth-dependent manner

**Fig 2:**
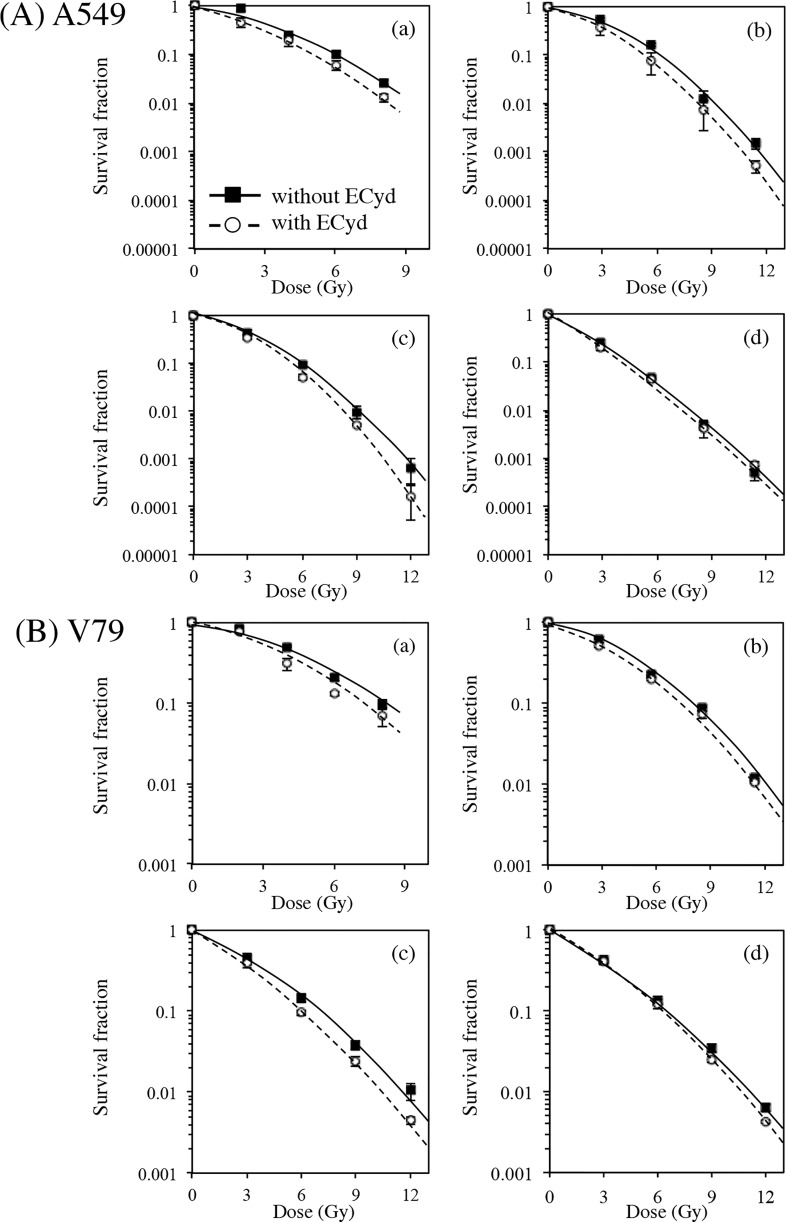
Clonogenic survival curves of cells treated with proton irradiation in the presence and absence of ECyd at different depths. Survival curves were obtained using A549 cells (A) and V79 cells (B). Each graph represents the results from different positions, (a)–(d).

**Fig 3:**
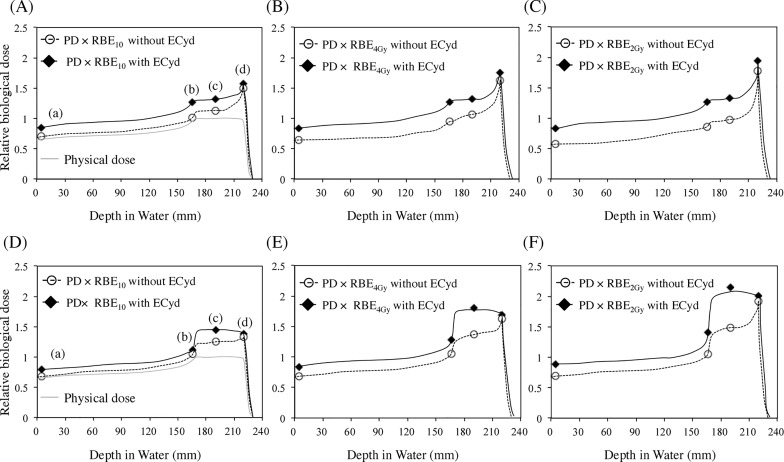
Relative dose-depth distributions. Dose-depth distributions in A549 cells (A)–(C) and V79 cells (D)–(F). Relative physical dose (PD) (gray solid line), PD × relative biological equivalent (RBE) in the absence of ECyd (white circle and dotted-line), PD × RBE in the presence of ECyd (black diamond shape and black solid line) at each depth ((a) through (d), as shown in Fig 1B) of the proton beam. The physical dose was normalized at the center of the spread-out Bragg peak. The values shown in S1 Table were used for RBE values [RBE_10_ for (A) and (D), RBE_4Gy_ for (B) and (E), REB_2Gy_ for (C) and (F)].

Fig 2 shows the results of the clonogenic assay for the surviving fraction of A549 and V79 cells exposed to proton beams at positions (a)–(d), as shown in Fig 1B. ECyd significantly enhanced proton-induced cell death in both A549 and V79 cells at positions (a), (b), and (c) and induced a small change in proton-induced cell death at position (d). These results indicate that ECyd was not effective at the distal edge (d) of SOBP. The biological parameters obtained from the survival curves are shown in Table 1. ECyd notably enhanced the α coefficient at positions (a), (b) and (c), and the alpha coefficient of the sensitizer enhancement ratio (SER_α_) ranged from 1.613 to 1.945 for A549 cells and from 1.443 to 1.791 for V79 cells at positions (a), (b) and (c). However, SER_α_ values for A549 and V79 cells at position (d) were 1.167 and 1.050, respectively. The β coefficient of SER (SER_β_) changed very little for both cell lines at all positions within SOBP. The ratio of the alpha and beta SER coefficients (SER_α/β_) at positions (a), (b) and (c) was higher than that at position (d) for each cell line. The biological doses, calculated for both cell lines by using RBE_10_, RBE_4Gy_, and RBE_2Gy_ values (S1 Table), are shown in Fig 3. ECyd prominently increased the biological dose at positions (b) and (c) but not at position (d) in SOBP, reducing the differences in biological dose within the regions of SOBP. SER_2Gy_ values at positions (b) and (c) were significantly higher than those of SER_10_ or SER_4Gy_, respectively. This observation suggests that the sensitizing effects of ECyd at positions (a), (b) and (c) are more remarkable at lower dose. In addition, LET_d_ values at each position are shown in Table 1. The LET_d_ value calculated at the distal edge (d) of SOBP was the highest and was more than twice as large as those at other positions.

**Table 1:** Survival parameters and sensitizer enhancement ratios for A549 cells and V79 cells irradiated with or without ECyd at various depth.

Cell line	Position	Depth (mm)	LET_d_ (keV/μm)	ECyd	α (Gy^-1^)	SER_α_	β (Gy^-2^)	SER_β_	α/β (Gy)	SER_α /β_
A549	a	5	0.857	-	0.127 ± 0.031	1.945	0.036 ± 0.026	1.083	3.498	1.912
A549	a	5	0.857	+	0.247 ± 0.059	1.945	0.039 ± 0.010	1.083	6.307	1.912
A549	b	165	2.847	-	0.143 ± 0.022	1.825	0.033 ± 0.005	1.061	4.275	1.743
A549	b	165	2.847	+	0.261 ± 0.129	1.825	0.035 ± 0.005	1.061	7.45	1.743
A549	c	190	3.695	-	0.160 ± 0.046	1.613	0.038 ± 0.008	0.895	4.233	1.884
A549	c	190	3.695	+	0.258 ± 0.048	1.613	0.034 ± 0.004	0.895	7.977	1.884
A549	d	220	9.457	-	0.420 ± 0.089	1.167	0.038 ± 0.014	0.868	17.404	1.442
A549	d	220	9.457	+	0.490 ± 0.173	1.167	0.033 ± 0.010	0.868	25.088	1.442
V79	a	5	0.857	-	0.097 ± 0.003	1.443	0.022 ± 0.003	1.136	4.582	1.227
V79	a	5	0.857	+	0.140 ± 0.008	1.443	0.025 ± 0.004	1.136	5.622	1.27
V79	b	165	2.847	-	0.091 ± 0.010	1.791	0.025 ± 0.004	0.88	4.037	1.887
V79	b	165	2.847	+	0.163 ± 0.011	1.791	0.022 ± 0.003	0.88	7.617	1.887
V79	c	190	3.695	-	0.160 ± 0.013	1.619	0.025 ± 0.003	0.84	6.555	1.969
V79	c	190	3.695	+	0.259 ± 0.026	1.619	0.021 ± 0.006	0.84	12.909	1.969
V79	d	220	9.457	-	0.242 ± 0.002	1.05	0.020 ± 0.001	1.05	12.123	1.068
V79	d	220	9.457	+	0.254 ± 0.005	1.05	0.021 ± 0.005	1.05	12.951	1.068

The biological parameters and sensitizer enhancement ratios (SER) were calculated based on survival curves.

### DNA double-strand breaks at the center of SOBP were repaired faster than those at the distal edge of SOBP

**Fig 4:**
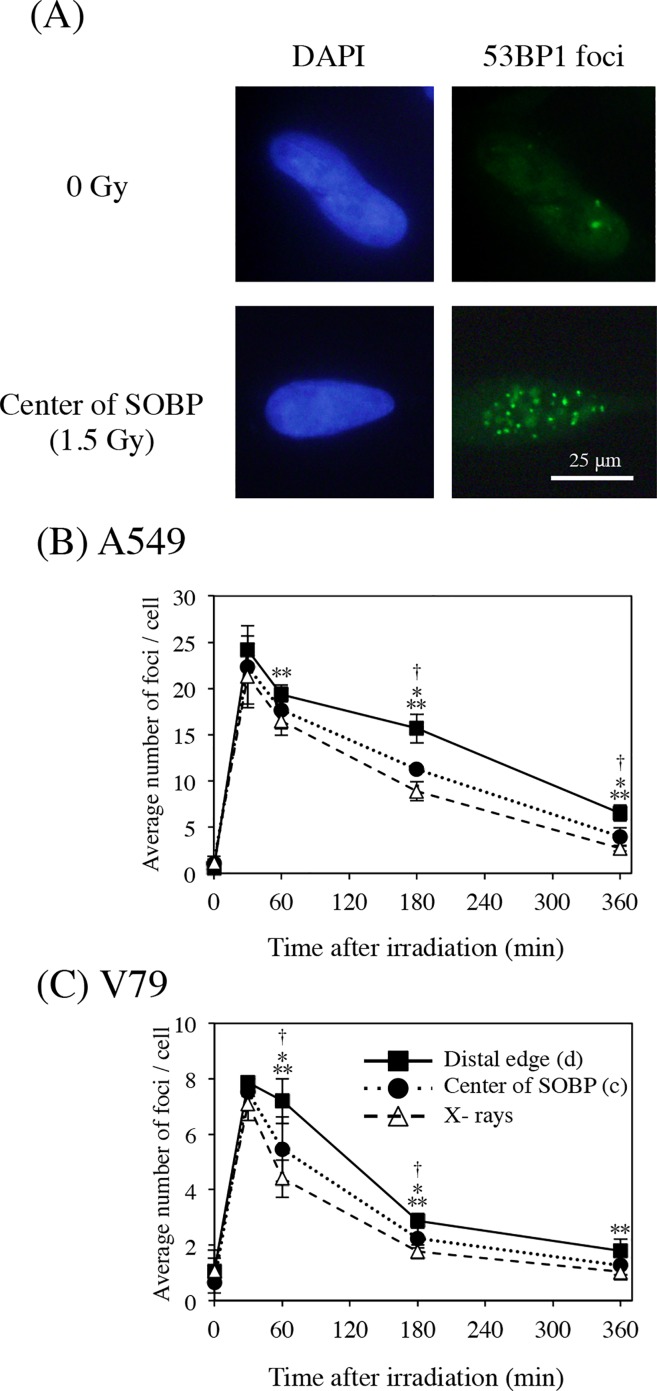
Comparison of DNA repair kinetics after proton- and X-irradiation. (A) Representative images of p53-binding protein 1 (53BP1) foci at the center of the spread-out Bragg peak (SOBP) in 4ʹ,6ʹ-diamidino-2-phenylindole (DAPI)-treated A549 cells 30 min after 1.5 Gy of proton irradiation. (B, C) Evaluation of DNA repair kinetics by counting 53BP1 foci formed in A549 cells (B) and V79 cells (C). After proton irradiation (1.5 Gy), cells were collected at the indicated times. The number of 53BP1 foci in at least 50 cells was scored and the average numbers were plotted. Data are expressed as mean ± S.D. from three experiments. *P < 0.05 for X-rays vs. the center of SOBP, **P < 0.05 for X-rays vs. the distal edge of SOBP and †*P* < 0.05 for the center vs. the distal edge of SOBP. Differences were evaluated using the Mann–Whitney U test.

To identify the reason for the significant difference in RBE values between the center and the distal edge of SOBP, the kinetics of DNA double-strand break (DSB) repair in A549 cells irradiated with proton beams or X-rays were evaluated at positions (c) and (d) in SOBP using the 53BP1 foci formation assay (Fig 4). Fig 4A displays representative photomicrographs revealing the proton-induced increase in 53BP1 foci of A549 cells. About 20 53BP1foci were clearly observed 30 min after 1.5 Gy of proton irradiation at the center (c) of SOBP. By counting the number of foci per cell at various times after proton irradiation at positions (c) and (d), the kinetics of DNA DSB repair was evaluated with respect to the difference in position within SOBP. As shown in Fig 4B, the average number of foci per cell at positions (c) and (d) 30 min after 1.5 Gy of proton irradiation peaked at approximately 20, although the rate of time-dependent decrease at the center (c) of SOBP was faster than that at the distal edge (d) of SOBP. With respect to these observations, the additional immunofluorescence staining for the γH2AX, which is used as well as 53BP1 as a biomarker of cellular response to DSBs widely (S2 Fig). A similar tendency was observed in the γH2AX formation assay and the rate of time-dependent decrease at the center (c) of SOBP was faster than that at the distal edge (d) of the SOBP. These results suggest that the structure of the DNA DSBs was easier to repair at the center (c) of SOBP that at the distal edge (d) of SOBP. In addition, there was no significant difference between the repair kinetics induced by proton irradiation at the center (c) of SOBP and those induced by X-irradiation.

Similar results were also observed with V79 cells (Fig 4C and S2 Fig).

### ECyd inhibited DSB repair at the center of SOBP, but not at its distal edge

**Fig 5:**
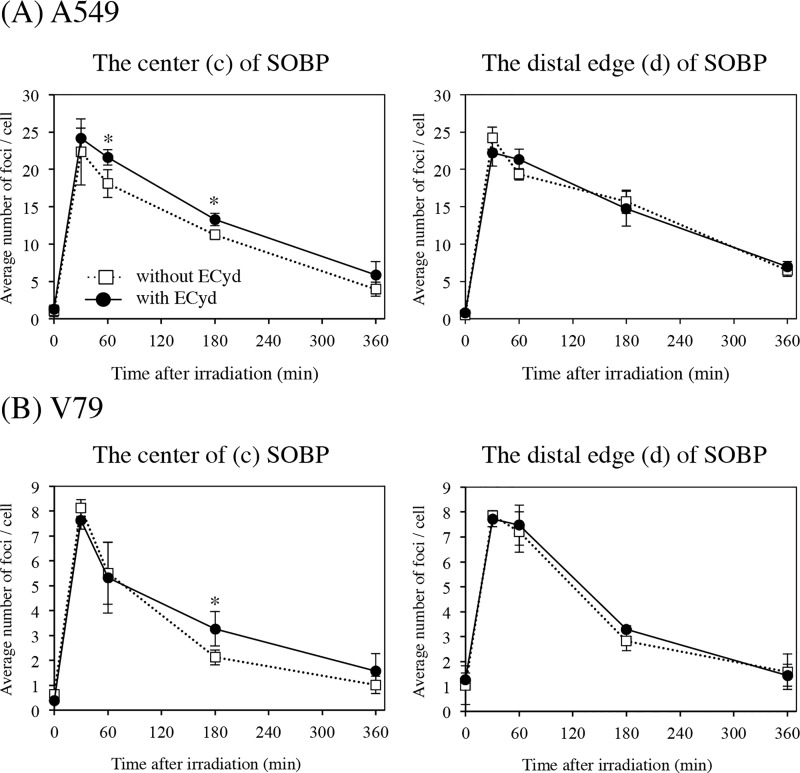
The depth-dependent effect of ECyd on DNA repair kinetics. Formation p53-binding protein 1 (53BP1) foci after proton irradiation at the center (c) and distal edge (d) of the spread-out Bragg peak (SOBP) in A549 (A) and V79 (B) cells. After treatment with 1.5 Gy of proton irradiation and/or ECyd, cells were collected at the indicated times. The number of 53BP1 foci in at least 50 cells was scored and the average numbers are plotted. Data are expressed as the mean ± S.D. from three experiments. *P < 0.05 (Mann–Whitney U test).

To understand the mechanism of the position-dependent radiosensitization induced by ECyd, the DSB repair kinetics in proton-irradiated cells cultured in the presence or absence of ECyd were evaluated at positions (c) and (d) in SOBP using the 53BP1 foci formation assay (Fig 5). Fig 5A shows results of the DSB repair kinetics obtained at positions (c) and (d) of SOBP in A549 cells. In the center (c) of SOBP 1 h and 3 h after irradiation, the number of 53BP1 foci from cells irradiated with ECyd were significantly higher than that from cells irradiated without ECyd, indicating that ECyd inhibited DSB repair at this position. Meanwhile, ECyd treatment did not induce any changes in the shape of the time-dependent decay curve for the number of foci at the distal edge (d) of SOBP, indicating that ECyd hardly affected DSB repair at the distal edge (d) of SOBP. Similar results were also observed with V79 cells (Fig 5B).

### ECyd enhances A549 cell senescence at the center of SOBP

**Fig 6:**
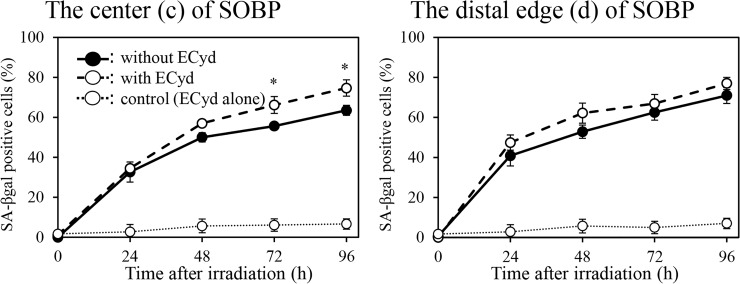
The depth-dependent effect of ECyd on radiation-induced cellular senescence. Quantification of cellular senescence at the center (c) and the distal edge (d) of the spread-out Bragg peak in A549 cells. After proton irradiation (9 Gy) and/or treatment with ECyd, cells were collected at the indicated times. The number of stained cells was scored among at least 200 cells, and the average numbers are plotted. Data are expressed as mean ± S.D. from three experiments. *P < 0.05 (Mann-Whitney U test).

As shown in Fig 2, treatment with ECyd increases reproductive cell death. To examine whether senescence, which is one of the factors that introduce reproductive cell death, is associated with the ECyd-induced radiosensitization of A549 cells at the center (c) of SOBP, a senescence-associated β-galactosidase assay was performed. The percentages of β-galactosidase positive A549 cells are shown in Fig 6. Treatment with ECyd only did not increase the number of senescent cells, which were not exposed to the proton beam, whereas treatment with ECyd increased the number of senescent cells at the center (c) of SOBP at 72 h and 96 h after irradiation. However, ECyd treatment did not result in a significant difference in the numbers of senescent cells at the distal edge (d) of SOBP. These results suggest that the induction of cellular senescence is at least partly associated with ECyd-induced radiosensitization in A549 cells exposed to the proton beam at the center (c) of SOBP.

## Discussion

In our previous study, clonogenic formation assays conducted using V79 cells demonstrated that RBE_37_ values at the center and the distal edge of SOBP formed during proton beam irradiation were 1.21 and 1.85, respectively [8]. Consistent with this observation, the present study showed significantly higher RBE values (1.9 times for A549 cells and 1.3 times for V79 cells) at the distal edge of SOBP (LET_d_ = 9.457 keV/μm) than at the center of SOBP (LET_d_ = 3.695 keV/μm), as shown in S1 Table.

A simulation study described by Scholz and Kraft demonstrated LET-dependence of cell killing by both heavy ions and protons [23]. In human fibroblast cell line AG01522, LET-dependence of cell killing was reported in proton beams with LETs in the range of 1–20 keV/μm [24] and there was a linear relationship between RBE and LET values of SOBP in proton beams [25]. Ward [26] and Goodhead [27] suggested that clustered DNA damage, which a cell is less able to repair, increases as LET increases. This clustered DNA damage is defined as two or more lesions formed within a few tens of base pairs by a single radiation track [28]. We examined the kinetics of DSB repair at the center (c) of SOBP and at the distal edge (d) of SOBP using the 53BP1 foci formation assay. As shown in Fig 4, there was no difference in the number of 53BP1 foci (23–24 foci per cell) 30 min after irradiation at these two positions within SOBP, suggesting that the initial number of DSBs at the distal edge (d) of SOBP was similar to that at the center (c) of SOBP. However, the decline in 53BP1-positive cells at the distal edge (d) of SOBP was slower than that at the center (c) of SOBP. A similar tendency was observed in the γH2AX formation assay (S2 Fig). In addition to counting the number of foci, the fluorescence intensity of 53BP1 foci was also measured (S3 Fig). The average intensity of foci per cell 30 min at the center (c) of the SOBP and the distal edge (d) of SOBP after irradiation reached the maximum value. However, the rate of time-dependent reduction of the intensity of foci at the center (c) of the SOBP was faster than that at the distal edge (d) of the SOBP as well as the rate of time-dependent decrease of the number of foci. This suggests that the amount of clustered DNA damage at the distal edge (d) of SOBP was larger than that at the center (c) of SOBP.

Next, the effect of ECyd on cell killing was examined at multiple positions of SOBP. SER_10_ was calculated at 10% survival (D_10_). SER_4Gy_ and SER_2Gy_ were calculated at D_4Gy_ and D_2Gy_, which are doses to induce same survivals of cells exposed to 250 kV X-rays of 4 Gy and 2 Gy. Fig 3 shows that ECyd enhanced cell killing in a depth-dependent manner in A549 cells and V79 cells. As shown in Fig 3, whether evaluated using D_10_, D_4Gy_, or D_2Gy_, ECyd treatment induced significant radiosensitization at positions (a), (b), and (c), but not (d). It was also observed in both cell lines that SER_2Gy_ values at positions (a), (b), and (c) were larger than SER_4Gy_ values at position (a), (b), and (c), respectively. To identify the mechanism responsible for this non-uniformity of ECyd-induced radiosensitization at different positions within SOBP, we examined the effect of ECyd on the DSB repair kinetics at the center (c) of SOBP and the distal edge (d) of SOBP using the 53BP1 foci formation assay. As shown in Fig 5, ECyd decelerates the decay rate of 53BP1 foci at the center (c) of SOBP but not at the distal edge (d) of SOBP. To confirm these observations, an additional foci formation assay using MDC1 antibody was performed in A549 cells. As shown in S4 Fig, in the center (c) of the SOBP 6 h after irradiation, the number of MDC1 foci from cells irradiated with ECyd was significantly higher than that from cells irradiated without ECyd and the same tendency of the 53BP1 foci formation assay was observed in the MDC1 foci formation assay. As mentioned above, these observations suggest that less repairable clustered DNA damage is concentrated at the distal edge (d) of SOBP compared with that at the center (c) of SOBP. Matsuda *et al*. reported that ECyd is cytidine analogue and rapidly undergoes phosphorylation to a 5ʹ- triphosphate form after its uptake into cells [14, 15]. This 5ʹ- triphosphate form strongly inhibits RNA polymerase to cause RNA synthesis inhibition. Therefore, the expression of various proteins is inhibited at the level of RNA synthesis. In previous studies about ECyd, it is reported that ECyd strongly suppresses the expression of anti-apoptotic proteins such as survivin [16–18] or TP53-induced Glycolysis and Apoptosis Regulator [29] and that ECyd inhibits hypoxia inducible factor 1α expression [17]. Moreover, Meike *et al*. demonstrated that ECyd treatment down-regulates *BRCA2* expression and consequently abrogates the homologous recombination pathway in A549 cells, HEp-2 cells, and V79 cells after X-ray irradiation [18]. Thus, the ECyd-induced sensitization at the center (c) of SOBP might be partly explained by ECyd-induced impairment of the DNA damage repair system, which is induced by the low LET component of the proton beam.

In a previous study, ECyd treatment was shown to induce radiosensitization through the induction of apoptotic cell death in Colon26 cells and MKN45 cells [16]. Morphological observation of nuclear fragmentation and condensation and the Annexin V/ propidium iodide-double staining technique were performed to examine whether ECyd enhances proton-induced apoptotic cell death. ECyd-induced enhancement of proton-induced apoptotic cell death was not observed at the center (c) of SOBP in either cell line (data not shown). However, as shown in Fig 6, ECyd significantly increased the number of proton-induced senescent A549 cells at the center (c) of SOBP, whereas ECyd treatment did not increase the number of proton-induced senescent A549 cells at the distal edge (d) of SOBP. Thus, the mode of death enhanced by ECyd-induced radiosensitization in this study was different that that reported previously. This may be due to the difference between X-rays and proton beams. Alternatively, this difference may be explained by cell line differences. Because ionizing radiation activates multiple signal transduction pathways, we are still far from understanding the complete network of interactions and regulatory mechanisms that decide whether a cell will choose one of the possible cell death pathways that to lead to apoptosis, mitotic catastrophe, autophagy, or senescence. Further study is necessary to clarify the mechanisms responsible for choosing each of cell death signaling pathways.

As shown in Fig 3 and S1 Table, ECyd reduced the difference between RBE at the distal edge (d) and that at the other positions ((b) or (c)) of SOBP in A549 cells and V79 cells, with the result that ECyd planarized RBE value of SOBP. Because a constant RBE value of 1.1 is conveniently used in clinical treatment planning for proton beam therapy, and LET-dependent nonuniformity of RBE in SOBP is sometimes ignored in clinical treatment, biological doses higher than the prescribed dose are deposited in the distal region of SOBP. This suggests that there is a risk of serious side effects at the distal margin of SOBP [7, 21, 25]. Therefore, ECyd treatment may be useful to evenly deposit the biological dose within the tumor tissue during proton therapy, by planarizing RBE within SOBP.

## Conclusion

In summary, we demonstrated that ECyd is an effective radiosensitizer for proton beams. SER at the center of SOBP was higher than that at the distal edge of SOBP because ECyd sensitized cell killing in a depth-dependent manner. Moreover, ECyd may be a suitable candidate to minimize the difference in RBE within SOBP of proton beams. The ECyd-induced planarization of the biological dose within SOBP may result in improved tumor control and reduce the risk of adverse effects at the distal edge of SOBP.

## Supporting Information

S1 Fig The chemical structure of 1-(3-C-ethynyl-β-D-ribo-pentofuranosyl) cytosine (ECyd). (PDF)

S2 Fig Comparison of DNA repair kinetics after proton- and X-irradiation. Evaluation of DNA repair kinetics by counting gamma H2AX (γH2AX) foci formed in A549 cells (A) and V79 cells (B). After proton irradiation (1.5 Gy), cells were collected at the indicated times. The number of γH2AX foci in at least 50 cells was scored and the average numbers were plotted. Data are expressed as mean ± S.D. from three experiments. *P < 0.05 for X-rays vs. the center of SOBP, **P < 0.05 for X-rays vs. the distal edge of SOBP and †*P* < 0.05 for the center vs. the distal edge of the SOBP. Differences were evaluated using the Mann–Whitney U test.
(PDF)

S3 Fig The measurement of the intensity of p53-binding protein 1 foci after proton- and X-irradiation. Evaluation of DNA repair kinetics by measuring of the intensity of 53BP1 foci formed in A549 cells (A) and V79 cells (B). After proton irradiation (1.5 Gy), cells were collected at the indicated times. The measurement of 53BP1 foci in at least 50 cells was scored and the average intensity was plotted. The average intensity at the indicated times was normalized at the value of 30 min after irradiation. Data are expressed as mean ± S.D. from three experiments. *P < 0.05 for X-rays vs. the center of SOBP, **P < 0.05 for X-rays vs. the distal edge of SOBP and †*P* < 0.05 for the center vs. the distal edge of SOBP. Differences were evaluated using the Mann–Whitney U test.
(PDF)

S4 Fig The depth-dependent effect of ECyd on DNA repair kinetics. Formation Mediator of DNA damage checkpoint protein 1 (MDC1) foci after proton irradiation at the center (c) and distal edge (d) of the spread-out Bragg peak (SOBP) in A549 cells. After treatment with 1.5 Gy of proton irradiation and/or ECyd, cells were collected at the indicated times. The number of MDC1 foci in at least 50 cells was scored and the average numbers are plotted. Data are expressed as the mean ± S.D. from three experiments. *P < 0.05 (Mann–Whitney U test).
(PDF)

S1 Table Summary of RBE_10_, RBE_2Gy_ and RBE_4Gy_ values. At D_10_ (the dose necessary to reduce the survival fraction to 10%), the values of the relative biological equivalent (RBE_10_) and the sensitizer enhancement ratio (SER) were calculated. Additionally, the RBE_2Gy_ and RBE_4Gy_ values were calculated at each position. RBE_2Gy_ and RBE_4Gy_ values were calculated as the ratio of the isosurviving fraction at 2 Gy or 4 Gy to that of X-rays. The D_10_ of X-rays was 6.85 Gy for A549 cells and 8.71 Gy for V79 cells.
(PDF)
