# Supplementary figures and images for: A Nucleoside Anticancer Drug, 1-(3-C-Ethynyl-β-D-Ribo-Pentofuranosyl)Cytosine, Induces Depth-Dependent Enhancement of Tumor Cell Death in Spread-Out Bragg Peak (SOBP) of Proton Beam
Source: PLoS One. 2016 Nov 22;11(11):e0166848. doi: 10.1371/journal.pone.0166848 (PMC5119790; doi:10.1371/journal.pone.0166848)

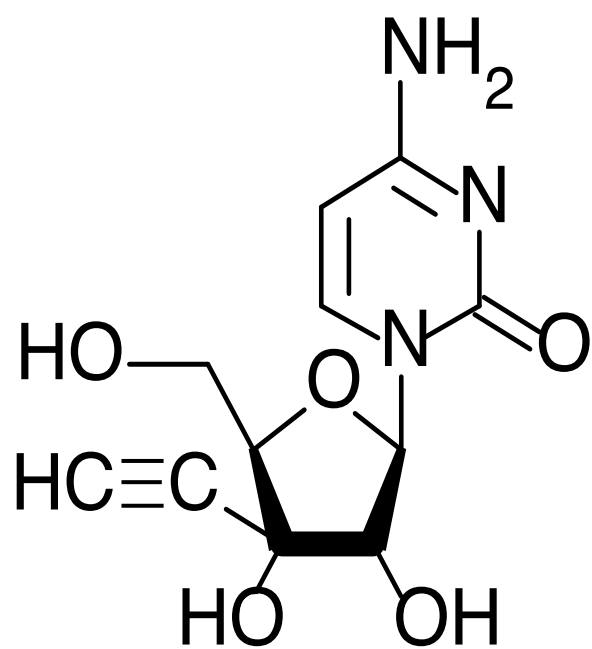

Supplement: S1 Fig — (PDF) [file pone.0166848.s001.pdf]

(A) A549

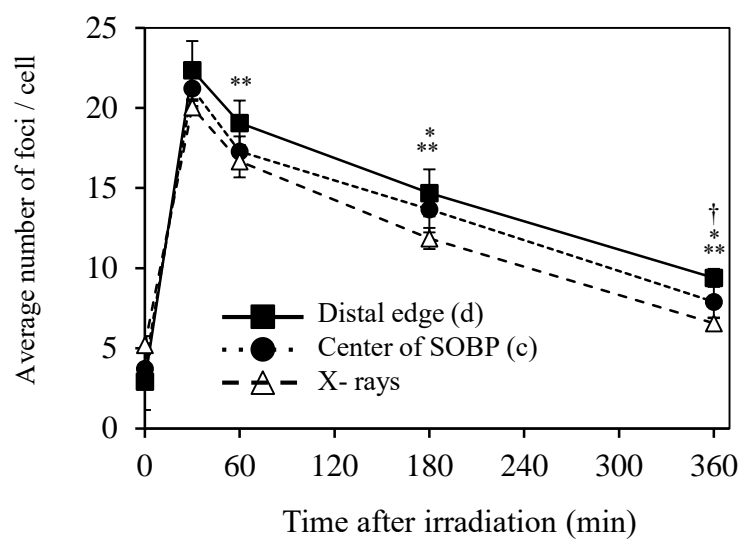

(B) V79

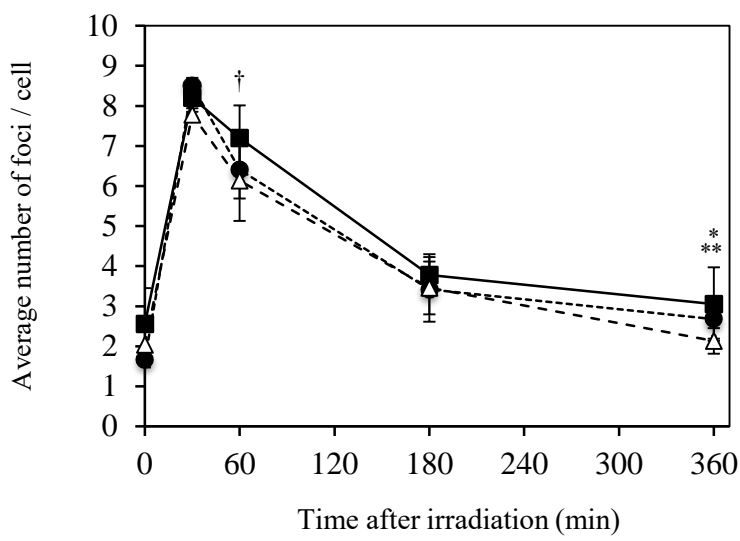

S2 Figure. Maeda *et al.*

Supplement: S2 Fig — Evaluation of DNA repair kinetics by counting gamma H2AX (γH2AX) foci formed in A549 cells (A) and V79 cells (B). After proton irradiation (1.5 Gy), cells were collected at the indicated times. The number of γH2AX foci in at least 50 cells was scored and the average numbers were plotted. Data are expressed as mean ± S.D. from three experiments. *P < 0.05 for X-rays vs. the center of SOBP, **P < 0.05 for X-rays vs. the distal edge of SOBP and †P < 0.05 for the center vs. the distal edge of the SOBP. Differences were evaluated using the Mann–Whitney U test. (PDF) [file pone.0166848.s002.pdf]

(A) A549

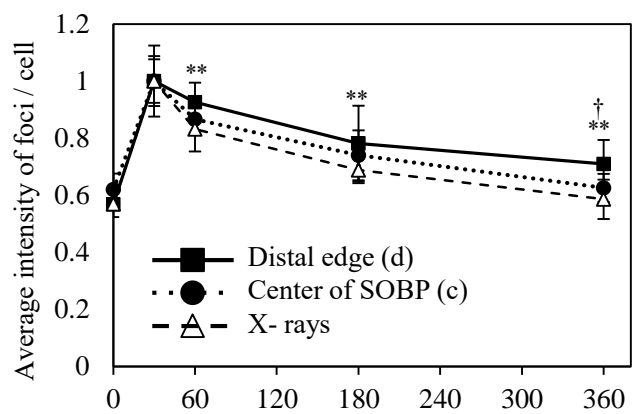

(B) V79

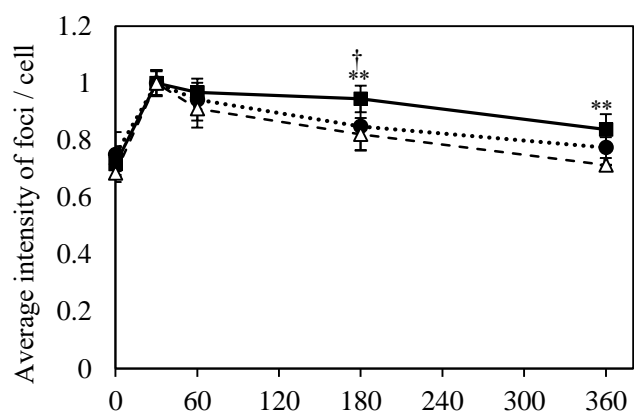

Supplement: S3 Fig — Evaluation of DNA repair kinetics by measuring of the intensity of 53BP1 foci formed in A549 cells (A) and V79 cells (B). After proton irradiation (1.5 Gy), cells were collected at the indicated times. The measurement of 53BP1 foci in at least 50 cells was scored and the average intensity was plotted. The average intensity at the indicated times was normalized at the value of 30 min after irradiation. Data are expressed as mean ± S.D. from three experiments. *P < 0.05 for X-rays vs. the center of SOBP, **P < 0.05 for X-rays vs. the distal edge of SOBP and †P < 0.05 for the center vs. the distal edge of SOBP. Differences were evaluated using the Mann–Whitney U test. (PDF) [file pone.0166848.s003.pdf]

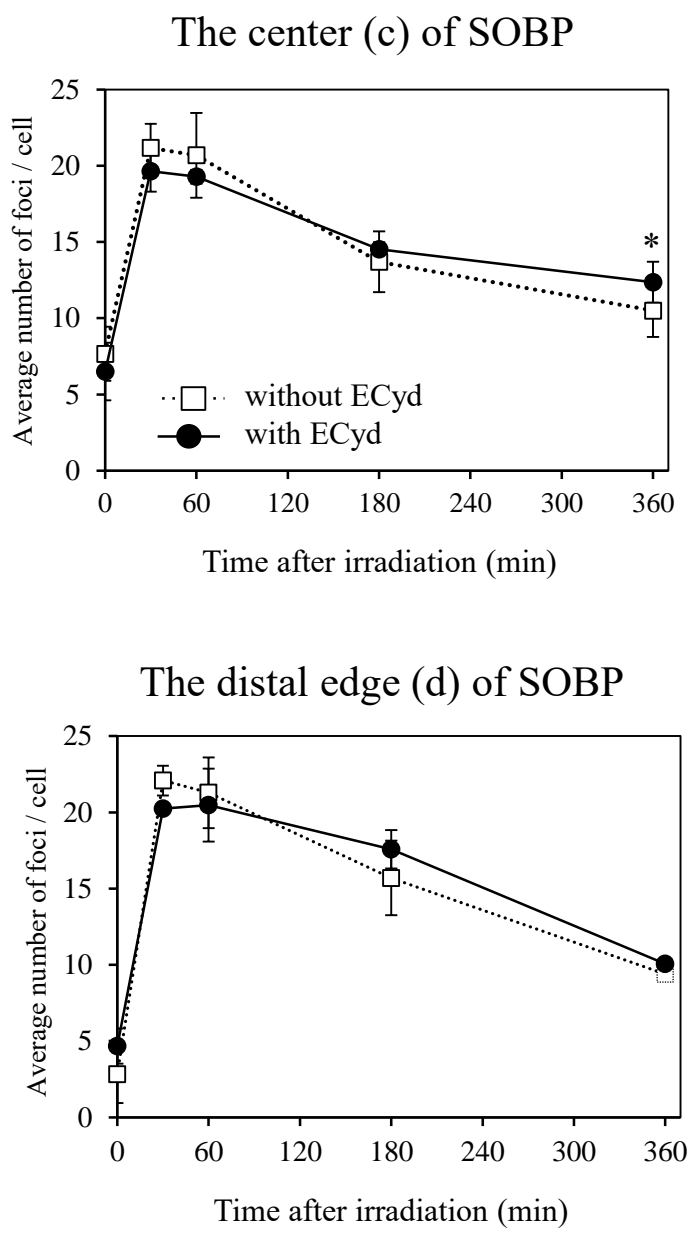

Supplement: S4 Fig — Formation Mediator of DNA damage checkpoint protein 1 (MDC1) foci after proton irradiation at the center (c) and distal edge (d) of the spread-out Bragg peak (SOBP) in A549 cells. After treatment with 1.5 Gy of proton irradiation and/or ECyd, cells were collected at the indicated times. The number of MDC1 foci in at least 50 cells was scored and the average numbers are plotted. Data are expressed as the mean ± S.D. from three experiments. *P < 0.05 (Mann–Whitney U test). (PDF) [file pone.0166848.s004.pdf]
