# Supplementary material for: A Nucleoside Anticancer Drug, 1-(3-C-Ethynyl-β-D-Ribo-Pentofuranosyl)Cytosine, Induces Depth-Dependent Enhancement of Tumor Cell Death in Spread-Out Bragg Peak (SOBP) of Proton Beam
Source: PLoS One. 2016 Nov 22;11(11):e0166848. doi: 10.1371/journal.pone.0166848 (PMC5119790; doi:10.1371/journal.pone.0166848)
Supplement: S1 Table — At D10 (the dose necessary to reduce the survival fraction to 10%), the values of the relative biological equivalent (RBE10) and the sensitizer enhancement ratio (SER) were calculated. Additionally, the RBE2Gy and RBE4Gy values were calculated at each position. RBE2Gy and RBE4Gy values were calculated as the ratio of the isosurviving fraction at 2 Gy or 4 Gy to that of X-rays. The D10 of X-rays was 6.85 Gy for A549 cells and 8.71 Gy for V79 cells. (PDF) [file pone.0166848.s005.pdf]

Supplementary Table 1. Summary of RBE_{10}, RBE_{2Gy} and RBE_{4Gy} values

Cell line	Position	Depth (mm)	LET_d (keV/μm)	ECyd	D_{10} (Gy)	RBE_{10}	SER	D_{4Gy} (Gy)	RBE_{4Gy}	SER_{4Gy}	D_{2Gy} (Gy)	RBE_{2Gy}	SER_{2Gy}
A549	a	5	0.857	-	6.414 ± 0.197	1.068 ± 0.051	1.209	4.087 ± 0.212	0.981 ± 0.052	1.293	2.300 ± 0.200	0.874 ± 0.077	1.418
				+	5.304 ± 0.531	1.299 ± 0.125		3.161 ± 0.453	1.282 ± 0.173		1.622 ± 0.325	1.264 ± 0.232	
	b	165	2.847	-	6.436 ± 0.181	1.065 ± 0.030	1.215	4.035 ± 0.092	0.992 ± 0.022	1.280	2.215 ± 0.098	0.904 ± 0.040	1.365
				+	5.296 ± 0.987	1.327 ± 0.277		3.153 ± 0.744	1.325 ± 0.360		1.623 ± 0.481	1.323 ± 0.460	
	c	190	3.695	-	6.021 ± 0.332	1.132 ± 0.062	1.157	3.763 ± 0.221	1.065 ± 0.065	1.239	2.059 ± 0.190	0.977 ± 0.093	1.360
				+	5.204 ± 0.230	1.316 ± 0.029		3.036 ± 0.127	1.319 ± 0.055		1.514 ± 0.105	1.326 ± 0.091	
	d	220	9.457	-	4.372 ± 0.206	1.568 ± 0.073	1.038	2.359 ± 0.255	1.709 ± 0.180	1.049	1.092 ± 0.176	1.863 ± 0.290	1.061
				+	4.211 ± 0.822	1.656 ± 0.257		2.249 ± 0.501	1.836 ± 0.392		1.029 ± 0.285	2.040 ± 0.527	
V79	a	5	0.857	-	8.439 ± 0.348	1.034 ± 0.046	1.167	3.836 ± 0.135	1.044 ± 0.036	1.225	1.904 ± 0.060	1.051 ± 0.033	1.290
				+	7.233 ± 0.567	1.211 ± 0.092		3.131 ± 0.230	1.282 ± 0.092		1.476 ± 0.102	1.359 ± 0.092	
	b	165	2.847	-	7.939 ± 0.671	1.102 ± 0.093	1.068	3.652 ± 0.307	1.100 ± 0.093	1.223	1.831 ± 0.155	1.098 ± 0.095	1.353
				+	7.433 ± 0.172	1.172 ± 0.031		2.987 ±0.088	1.340 ± 0.040		1.353 ± 0.056	1.480 ± 0.061	
	c	190	3.695	-	6.947 ± 0.112	1.254 ± 0.020	1.157	2.928 ± 0.043	1.366 ± 0.020	1.340	1.348 ± 0.056	1.486 ± 0.060	1.440
				+	6.003 ± 0.419	1.456 ± 0.127		2.229 ± 0.161	1.801 ± 0.123		0.936 ± 0.077	2.146 ± 0.176	
	d	220	9.457	-	6.263 ± 0.170	1.392 ± 0.073	1.038	2.352 ± 0.103	1.702 ± 0.073	1.040	0.993 ± 0.053	2.017 ± 0.108	1.037
				+	6.031± 0.152	1.445 ± 0.165		2.262 ± 0.202	1.778 ± 0.165		0.958 ± 0.122	2.111 ± 0.281	

RBE_{10}, RBE_{4Gy} and RBE_{2Gy} were calculated as the ratio of 10% survival fraction (D_{10}) and isosurviving fraction at 4 Gy (D_{4Gy}) and 2 Gy (D_{2Gy}) to that of 250 kV X-rays, respectively. D_{10}s of X-rays were 6.8[illegible] Gy and 8.71 Gy for A549 cells and V79 cells, respectively.
